# 

**Published:** 1890-06

**Authors:** 


10 cts. a copy. JUNE, 1890. $1.00 per year.

### HALL'S JOURNAL OF HEALTH

ESTABLISHED 1854

OFFICE, 206 BROADWAY, EVENING POST BUILDING, Room 11, N. Y.

Entered as Second-Class Matter at the Post-Office, New York.

